# Post-COVID-19 Condition: How Sociodemographic Factors, Physical Well-Being and Functionality Influence Quality of Life and Mental Health Symptoms

**DOI:** 10.3390/healthcare12151551

**Published:** 2024-08-05

**Authors:** Mᵃ Pilar Rodríguez-Pérez, Marta Pérez-de-Heredia-Torres, Pilar Rodríguez-Ledo, Gemma Fernández-Gómez, Cristina García-Bravo, Roberto Cano-de-la-Cuerda, Patricia Sánchez-Herrera-Baeza

**Affiliations:** 1 Department of Physical Therapy, Occupational Therapy, Physical Medicine and Rehabilitation, Rey Juan Carlos University, 28922 Alcorcón, Spain; pilar.rodriguez@urjc.es (M.P.R.-P.); gemma.fernandez@urjc.es (G.F.-G.); cristina.bravo@urjc.es (C.G.-B.); roberto.cano@urjc.es (R.C.-d.-l.-C.); patricia.sanchezherrera@urjc.es (P.S.-H.-B.); 2 Research Group in Evaluation and Assessment of Capacity, Functionality and Disability (TO + IDI), Health Sciences Faculty, Rey Juan Carlos University, Avenida de Atenas s/n, 28922 Alcorcón, Spain; 3 Long COVID Working Group of the Spanish Society of General and Family Physicians (SEMG), Spanish Society of General Practitioners and Family Doctors (SEMG), Spanish Research Network on Long COVID (REiCOP), Management of the Health Area of Lugo, A Mariña and Monforte de Lemos, 27002 Lugo, Spain; prodriguezl@semg.es; 4 Research Group, Motion Analysis, Biomechanics, Ergonomy and Motor Control Laboratory (LAMBECOM), Faculty of Health Sciences, Rey Juan Carlos University, Alcorcón, 28922 Madrid, Spain

**Keywords:** long COVID, function, persistent symptoms, fatigue, quality of life, mental health

## Abstract

Background: Long COVID-19 syndrome remains a global public health problem, with more than 145 million people affected with multisystemic symptoms. Addressing the requirements of individuals impacted by a syndrome characterised by a complex and variable clinical presentation is of utmost importance. Identifying the variables that can exert influence and understanding their progression is essential for directing treatment strategies aimed at enhancing both independence and quality of life. Therefore, the aim of this study was to analyse the influence of sociodemographic and clinical variables on existence and their relationship with asthenia, anxiety symptoms and low mood. Methods: An analytical study secondary to an observational cross-sectional descriptive study. Results: Logistic regression showed significant univariate effects on asthenia [sex (*p* = 0.034); age (*p* = 0.042); Activities of Daily Living Questionnaire [ADQL (*p* = 0.002)] [physical functioning (*p* < 0.001) and general health (*p* = 0.014)] and multivariate [sex (*p* = 0.019), adult age (*p* = 0.01) and physical functioning (*p* = 0.04)]]; low mood [time of evolution (*p* = 0.028) and multivariate [time course (*p* = 0.007), ADLQ (*p* = 0.011), role physical (*p* = 0.013) and general health (*p* = 0.001)]] and anxiety [physical functioning (*p* = 0.046) and multivariate [physical functioning (*p* = 0.034), age (*p* = 0.011), time of evolution (*p* = 0.001) and ADQL (*p* = 0.011)]]. Conclusions: Increased age, gender and longer evolution time seem to favour the prevalence and occurrence of mental health symptoms; greater independence and good physical functioning are protective factors with respect to the occurrence of mental health-related symptoms in patients affected by post-COVID-19 condition.

## 1. Introduction

Acute COVID-19 infection causes a characteristic clinical picture of symptoms such as fever, malaise, headache, and fatigue that, in some cases, becomes persistent [1]. The World Health Organization (WHO) has established a clinical definition of persistent COVID-19 by international Delphi consensus. “Post-COVID-19 is the condition occurring in individuals with a history of probable or confirmed SARS-CoV-2 infection, usually 3 months after onset, with symptoms that last at least 2 months and cannot be explained by an alternative diagnosis. Common symptoms include, but are not limited to, fatigue, shortness of breath and cognitive dysfunction, and generally have an impact on daily functioning. Symptoms may be new onset after initial recovery from an acute episode of COVID-19 or persist from the initial illness. Symptoms may also fluctuate or relapse over time” [2]. Current data on the impact of the disease refer to more than 145 million people affected worldwide [3]. Between 11% and 24% of people affected by COVID-19 may experience long-term symptoms [4]. These symptoms have been identified independent of the acute severity of COVID-19. In fact, the prevalence of mild disease is 80% [5]. Despite this high percentage, it must be considered that these people, who were not hospitalised in the acute phase of COVID-19, suffered a disadvantage in healthcare compared to other diseases due to restrictions and isolation measures. In Spain, social restrictions continued, especially with the most vulnerable population, until two years after the start of the pandemic. This makes them a vulnerable population that requires greater attention and study [6].

Regarding the profiles of post-COVID-19 sufferers, the literature reports that they are predominantly female, with an average age of 43 years and no previous major health problems, lacking, in most cases, the presence of comorbidities involving physical difficulties and no diagnosis of depression or anxiety prior to COVID-19. Most of the cases went through an acute phase of COVID-19 disease, generally mild and without hospitalisation; therefore, the follow-up of their acute phase was on an outpatient basis [7,8]. The hypothesised aetiopathogenesis of persistent COVID-19 is that the mechanisms of this virus may lead to long-term tissue damage, neuroinflammation and alterations in the autoimmune system. This may explain why patients with post-COVID-19 condition may present with these persistent symptoms that fluctuate over time and most of the time without a clear recovery from the onset of acute COVID-19 [9]. Among the most frequent symptomatology that persists or develops residually, neurological and psychological symptoms are described, including depression, anxiety, cognitive impairment, asthenia, apathy and sleep disorders, among others. These variables are critical indicators of negative mental health, often used in clinical assessments due to their significant influence on quality of life and daily functioning [10]. The presence of significant chronic persistent asthenia in this population is also noteworthy, characterised not only by physical fatigue but also by a lack of vitality and energy, as well as mental fatigue, that interfere with daily life. Additionally, these patients often experience a pronounced worsening upon engaging in physical exercise, known as post-exertional malaise (PEM) [10,11]. However, the available evidence still reflects confusion, as it is not entirely clear whether these mental health symptoms persist as such due to the neurobiological impact of the disease itself or develop over time due to the influence of other factors that need to be studied [11]. Different investigations have primarily focused on analysing the impact of physical symptoms and their repercussions on functioning and performance in daily activities. Physical functioning, self-perception of physical health and the performance of physical roles go beyond mere symptoms but, rather, relate to an individual’s capacity and perception to perform daily physical activities compared to their pre-disease situation, and these factors may contribute to worsening emotional well-being [12,13]. Recent systematic reviews, such as the one conducted by Pizarro et al. [14], have concluded that individuals with post-COVID-19 condition experienced worsening and deterioration in performing activities of daily living (ADL) compared to their situation before the COVID-19 diagnosis, and this inability to perform ADLs or dependence on others to perform them can significantly affect a person’s perception of quality of life [15]. It is therefore necessary to understand the impact of these variables on outpatients. Due to their overlapping nature, their diagnostic criteria are often confused, and this highlights their interrelated effects. Understanding their interactions provides a global view of the dynamics of mental health in post-COVID-19 patients, facilitating targeted interventions and prioritising intervention at the right time, focusing especially on home-based support [14,16]. Authors such as Huang et al. and Poudel et al. [15,17] have demonstrated improvements in the independence of post-COVID-19 patients as time progresses; however, they are not sufficient, as they continue to experience low levels of HRQoL far from their pre-disease perception. Other authors such as Rodríguez-Galán et al. [16] have reinforced these findings, indicating that these mental health symptoms increase as the time since infection is longer. This neuropsychological syndrome occurs in almost one-third of those affected, immediately after the acute phase of COVID-19, and its prevalence progressively increases in the long term [10]. Consequently, the repercussions and impact on mental health are considerable, with some authors even stating that we are experiencing a psychiatric epidemic due to COVID-19 [18]. Therefore, this syndrome continues to be a significant global public health problem that affects both patients and society in the long term. It is necessary to establish evidence and analyse the impact to implement appropriate interventions and promote the improvement and well-being of those affected [13]. By focusing on these variables, our study aims to provide a nuanced understanding of the dynamics of negative mental health in long COVID-19 patients, offering insights that could inform targeted interventions and improve patient outcomes.

Therefore, the aim of this study was to analyse the influence of sociodemographic variables, the effect of physical health related to HRQoL and the degree of dependence on mental health symptom clinical variables. Our initial hypothesis is that variables such as age, gender and disease duration, as well as clinical variables like physical functioning, bodily pain and performance of role physical, general health (HRQoL) and dependency status could influence the prediction of asthenia, anxiety and low mood in individuals with post-COVID-19 condition.

## 2. Materials and Methods

### 2.1. Design

A secondary analysis was conducted as part of a cross-sectional descriptive study involving a cohort of Spanish adult patients diagnosed with post-COVID-19 syndrome [19]. Adherence to the Strengthening the Reporting of Observational Studies in Epidemiology (STROBE) checklist was ensured, which provides guidelines for comprehensive and accurate reporting in observational studies [20]. Approval for this study was obtained from the ethics committee of the Rey Juan Carlos University under reference number 170120210212. Prior to participation, everyone provided informed consent by signing a consent form.

### 2.2. Sample

Data collection occurred between April and July 2021, utilising videoconference surveys with individuals affected by long COVID. The Spanish authorities still had mobility restrictions in place for the most vulnerable people. The criteria for sample selection and variables were established in collaboration with the Spanish Society of General and Family Physicians (SEMG), drawing from previous descriptive studies that outlined the characteristics of the most affected population [21]. A calculation of the sampling parameter, based on an estimation, was performed to obtain an adequate power of the inferences. The software G * Power (version G * Power 3.1.9.2) was used, resulting in a sample size requirement of 120 participants. The inclusion criteria were meticulously chosen to ensure the representation of individuals most likely to manifest persistent symptoms after acute COVID-19 infection. These criteria encompassed individuals aged 30–50 years, diagnosed with acute-phase COVID-19 by polymerase chain reaction (PCR) and/or positive serology yet not requiring hospitalisation during this phase and no previous pathologies or comorbidities. Individuals had to have a medical diagnosis of post-COVID-19 syndrome according to the international Delphi consensus. In addition, they had to have multiple and persistent COVID-19 symptoms for three months or more. The mental health symptoms had to be confirmed by medical diagnosis and could not be attributed to other comorbidities or previous COVID-19 diseases. In addition, they were reflected in their medical history with the date of onset of each symptom. These symptoms were verified in the online survey and in the clinical interview, quantifying their occurrence, frequency and intensity. Patients had to have adequate communication skills to be able to understand and conduct the clinical interview. At the beginning of these interviews, the participant’s communication skills were assessed, ensuring that they were able to understand the questions and provide detailed and coherent answers.

Exclusion criteria were carefully considered to prevent confounding factors; for instance, individuals undergoing rehabilitation treatment for COVID-19 at the time of evaluation were excluded to avoid interference with symptom presentation. Furthermore, individuals lacking the necessary technology for the interview were excluded to ensure uniform data collection methods. The requirement for participants to accept and sign the informed consent form emphasised the ethical considerations inherent in the research process. These stringent criteria were selected to foster a cohort that accurately reflects the target population and to maximise the validity and reliability of the study findings.

### 2.3. Procedure

The project was conducted in collaboration with the SEMG, along with representatives from the Autonomous Communities “Long COVID-19 Together Spain” (ACTS). Both entities conveyed and disseminated information to individuals affected by the post-COVID-19 condition who had received the diagnosis from their primary care and specialist physicians. Following this process, patients who expressed interest in and willingness to participate in the research were registered in the online survey. The self-report questionnaire collected contact information; sociodemographic and clinical variables such as age, sex, method of diagnosis of COVID-19 during the acute phase, date of diagnosis, presence of general and mental health symptoms post-COVID-19 by specialist physician diagnosis and confirmation of participation in the study. Upon completion of the questionnaire and implicit agreement to the informed consent, the researcher proceeded to conduct a videoconference interview with the participant.

During the interview, a clinical history was taken, rechecking the data that the participant had completed on the form, and the corresponding assessment scales Activities of Daily Living Questionnaire (ADLQ) and SF-36 Health Questionnaire were added and administered. ADLQ was administered by inquiring about their current condition during the interview, followed by repeating the same questions about their condition before the onset of the disease. Subsequently, the anonymised data were archived in a digital booklet available only to the principal investigator.

### 2.4. Measures

ADLQ [22] is an assessment tool that can be self-administered to the patient or caregiver and measures the functional capacity of patients in relation to their different activities of daily living. It measures functioning in six performance areas: self-care, home care and management, employment and leisure, shopping and money management, transportation and communication. This scale is composed of 28 items that are scored from 0 (no problem) to 4 (can no longer perform the activity). Total scores and subscales were expressed as a percentage to indicate the degree of dependency. The degree of impairment was classified as “severe” (>66%), “moderate” (34–66%) or “none to mild” (0–33%). Satisfactory psychometric properties of the ADLQ scale have been demonstrated, and it has been validated and adapted to the Spanish population [23].

The impact on HRQoL was measured using the SF-36 Health Survey Questionnaire [24]. It consists of 36 items that can be self-administered or interview-based and measures domains related to physical functioning, role physical, bodily pain, general health, social functioning, role emotional, mental health and, finally, health transition. All dimensions refer to the current state compared to the pre-illness state. The domains of physical functioning in health-related quality of life, as measured by the SF-36, are dimensions that assess a person’s ability to carry out physical activities and their perception of their own physical health status. These domains encompass aspects such as the ability to engage in vigorous activities, the ability to perform moderate activities, the ability to carry out daily activities, the perception of one’s own physical health and the perception of changes in physical health over a specified period. Each item is rated on a scale of 0 to 100, with 0 being the lowest score and 100 the highest possible score. A higher score indicates a better health status. In this study, version 1 of the SF-36 and the “standard” version of the time frame were used, with a recall period of 4 weeks. To derive the PCS and MSC values, we extracted scores for each of the 8 domains and standardised them using a z-score transformation. These scores were subsequently multiplied by 10 and then added to 50 to produce normalised scores for each domain. Aggregation was achieved using factor score coefficients, resulting in normalised scores for each component [25]. This instrument is psychometrically robust and has been validated and adapted for the Spanish-speaking population [26].

A self-report questionnaire and semi-structured clinical interview were used to collect post-COVID-19 symptoms, taking into account the presence or absence of symptoms, intensity and impact of symptoms on daily life.

### 2.5. Data Analysis

Indicators were selected to assess the possible influence of various factors on the presence or absence of asthenia, anxiety or low mood. Specifically, these indicators were chosen to examine the impact of sociodemographic variables (age, gender, and time since diagnosis) and clinical variables, such as dependency status and physical functioning, at the current time of post-COVID-19. Descriptive statistics were used to characterise qualitative and quantitative variables, while logistic regression models were used to predict symptoms of asthenia, anxiety or depression at both the univariate and multivariate levels. Statistical analyses were conducted using SPSS 27.0 for Windows (Copyright© 2013 IBM SPSS Corp., Armonk, NY, USA), with statistical significance defined as *p* < 0.05.

## 3. Results

The study sample included 122 participants from various Spanish regions, all exhibiting persistent, multisystemic symptoms. Among them, 77.9% (n = 95) were women and 22.1% (n = 27) were men, aged 30 to 50 years, with an average age of 43.5 years (SD = 5.8). Participants reported physical and mental fatigue, low mood (56.9%, n = 70) and anxiety (43.9%, n = 54), with symptoms impacting daily life and functionality. Table 1, Table 2 and Table 3 provide a descriptive summary of the analysed demographic and clinical variables.

Participants showed an average level of dependency (ADLQ_TOTAL = 31.37 ± 9.65) and low health-related quality of life according to SF-36 subscale scores: the physical component summary (PCS) was 24.66 (SD = 4.45), physical functioning 27.50 (SD = 20.40), role physical 5.12 (SD = 16.99), general health 29.51 (SD = 16.23) and bodily pain 36.52 (SD = 22.04). The study protocol is detailed elsewhere [19]. The clinical variables were analysed for the presence of asthenia, using univariate and multivariate logistic regression models to determine their predictive effects.

In Table 1, univariate analysis showed significant effects for the sex (*p* = 0.034), age (*p* = 0.042), ADQL (*p* = 0.002) and SF-36 dimensions of physical functioning (*p* < 0.001) and general health (*p* = 0.014). Multivariate analysis revealed that females were 6.45 times more likely to experience asthenia than males (*p* = 0.019). Increased age (*p* < 0.001) and reduced physical functioning (*p* = 0.004) were also significant predictors of asthenia. Being female (*p* = 0.019), older age (*p* = 0.01) and reduced physical functioning (*p* = 0.004) maintained their significance, while ADQL and SF-36 general health no longer showed significant effects.

Table 2 presents the results of the logistic regression models for predicting low mood, both at the univariate and multivariate levels. In the univariate analysis, the time of evolution was the only variable with a significant effect (*p* = 0.028). In the multivariate analysis, time of evolution remained significant (*p* = 0.007), indicating that a longer duration of symptoms increases the likelihood of experiencing low mood. Additionally, the state of dependence measured by ADLQ (*p* = 0.011) and the SF-36 dimensions of role physical (*p* = 0.013) and general health (*p* = 0.001) also showed significant effects. Specifically, higher ADLQ scores, which indicate greater independence, were associated with a lower probability of low mood. Similarly, higher scores in the SF-36 dimensions of role physical and general health were linked to a decreased likelihood of experiencing low mood.

Regarding anxiety, Table 3 presents the descriptive results and the logistic regression models for predicting anxiety at both the univariate and multivariate levels. In the univariate analysis, physical functioning was the only variable with a significant effect (*p* = 0.046). In the multivariate analysis, physical functioning remained significant (*p* = 0.034), and additional variables such as age (*p* = 0.011), time of evolution (*p* < 0.001) and ADQL (*p* = 0.011) also showed significant effects. Specifically, as age increases—particularly, nearing 50 years—the likelihood of experiencing anxiety symptoms increases. Similarly, a longer duration of symptoms is associated with a higher probability of anxiety. Conversely, higher ADLQ scores, indicating better independence and better physical functioning, are both linked to a lower probability of experiencing anxiety symptoms.

## 4. Discussion

The aim of the present study was to analyse the influence of sociodemographic and clinical variables such as dependency status and physical health in relation to their quality of life and their possible influence on the existence and/or appearance of symptoms of asthenia, anxiety or depression. These findings suggest that symptoms such as anxiety and depression are more likely to develop and become prevalent over time, aligning with our study’s focus on the temporal emergence of these symptoms rather than their severity.

In terms of gender-related findings, our results are in line with the existing literature and, further, underscore the predominance and heightened impact of persistent COVID-19 within the female demographic. Previous research has identified sex-based disparities in mental health symptoms stemming from the COVID-19 pandemic [27]. Similarly, Sykes et al. [28], in their descriptive investigation of post-COVID-19 patients, concluded that women exhibited significantly higher occurrences of anxiety, low mood, physical fatigue or sleep disturbances. However, they did not provide a comprehensive examination of symptoms such as asthenia from a holistic perspective. The study by Calabria et al. [11] indicated that gender could serve as a predisposing factor for fatigue and highlighted a robust correlation between asthenia and apathy. Our study corroborates these findings, demonstrating that affected individuals experienced both physical and mental fatigue. Nevertheless, unlike previous research, our study delved into more detailed analyses of other specific psychiatric symptomatology, enhancing the breadth and relevance of our findings compared to previous investigations.

In our analysis, age emerged as a key factor predicting the development of apathy-asthenia and anxiety. Our findings suggest a tendency for these symptoms to manifest as individuals approach the age of 50. Previous research by Calabria et al. and Neufeld et al. [11,12] has underscored the prevalence of chronic fatigue, both physical and mental, among post-COVID-19 individuals, particularly in middle-aged cohorts. Our study extends these observations by highlighting an increased susceptibility to anxiety symptoms as well. However, the findings of Bucciarelli et al. [18], which indicated a higher prevalence of psychiatric symptoms in women under 50, may appear contradictory. This discrepancy can be explained by previous studies categorising post-COVID-19 cases by age, revealing a diminishing likelihood of such symptoms beyond the age of fifty. Our study’s significant results align closely with the mean age of the post-COVID-19 cohort under examination. Moreover, existing evidence consistently identifies the age range of 43 to 46 years as the most affected demographic [8,10,18].

Regarding the relationship between dependency status and symptoms of low mood and anxiety, our findings indicate that individuals with a higher level of dependency are more likely to experience these symptoms. These findings suggest that individuals affected by post-COVID-19 syndrome continue to face challenges in returning to their pre-illness normal life, impacting their autonomy and independence, thus potentially increasing the likelihood of experiencing symptoms of depression or anxiety. In this context, a systematic review by de Olivera et al. [29] concluded that individuals with persistent COVID-19 exhibited decreased performance in daily activities, leading to a loss of autonomy and independence with a negative impact on self-perceived quality of life. However, the studies reviewed focused on hospitalised patients in the acute phase and did not extend beyond six months post-COVID. Other previous studies, such as [30], also examined the long-term limitations in daily activities of patients with prolonged COVID-19 who were hospitalised during the acute phase [7], their findings aligning with ours. Given the significant percentage of outpatient individuals currently experiencing post-COVID-19 condition, analysing this profile becomes particularly relevant, as done exclusively in our research. Authors like Poudel et al. [17] have concluded that this population continues to exhibit poorer HRQoL compared to their pre-illness state and similar populations, suggesting that, despite slight improvements in long-term daily activities since the COVID-19 diagnosis, they still perceive their health poorly compared to before, contributing to a lack of mental well-being. However, to date, we have not found any studies that focused on previously non-hospitalised post-COVID-19 patients, analysing the relationship between limitations in independence and mental health. This could be highly relevant, since, beyond the impact of the disease symptoms themselves, independence in daily activities can be a protective factor and indicate better mental health conditions. Therefore, our data are relevant for the design of targeted intervention programs focused more on functionality and environmental adaptation to promote well-being and prevent mental health impacts in patients.

The association between poorer physical functioning and role physical favoured a predisposition toward symptoms such as anxiety and low mood. In this context, several population-based studies have demonstrated a strong correlation between decreased physical fitness and the likelihood of developing anxiety or depression disorders in the general population [31]. However, beyond physical well-being, it is intriguing to explore an individual’s capacity to engage in physical activities, as well as how their physical role in daily life may be affected compared to their pre-illness status, as investigated in this study. Previous studies like that of Premraj et al. [10] have also highlighted the prevalence of mental health symptoms in patients affected by post-COVID-19, along with their impact on physical and social functioning, suggesting that factors such as restriction, stress or disease management may also contribute. These authors primarily focused on symptomatology and its singular impact. More recent findings have linked the effects of fatigue, a common symptom in post-COVID-19 condition, to long-term physical and psychosocial functioning limitations, as well as its association with the development and prediction of mental health symptoms such as anxiety, depression and sleep disorders [11]. While their results mirrored those of this study, they were limited to patients admitted to the ICU during the acute phase of COVID-19 and its exclusive association with fatigue. No similar research has been found examining the relationship between physical functioning and mental health symptoms in non-hospitalised patients with long COVID; our findings suggest that individuals with lower scores in the PCS of HRQoL are a predisposing factor for worse mental health conditions. Thus, this research may contribute to guiding the design of early rehabilitation programs and taking a holistic approach with individuals with the aim of preventing potential impacts on mental health conditions.

Ultimately, one of the most pertinent findings of our study is the demonstrated correlation between the duration of persistent symptoms, such as anxiety and low mood. Our analysis reveals a notable increase in the prevalence of anxiety and depression symptoms over the duration of the illness, indicating a temporal relationship rather than an assessment of symptom severity. However, the underlying cause of the delayed onset of these symptoms still warrants investigation in future studies. From a neurobiological perspective, prior research has suggested a convergence of underlying mechanisms contributing to central nervous system dysfunction, including neuroinflammatory effects and autoimmunity, among others [9]. The previous literature indicates that a significant subset of individuals with post-COVID-19 mental health symptom conditions also exhibit signs of chronic inflammation [32,33]. Despite the lack of extensive research on the post-COVID-19 population, it remains unclear whether the persistence or onset of these neurological disorders is directly attributable to the inflammatory processes of SARS-CoV-2 or if other factors play a contributory role, such as the overall experience of illness and post-COVID-19 recovery and evolution [33]. Premraj et al. [10] concluded in their meta-analysis that over the medium to long term, individuals with post-COVID-19 syndrome exhibit a higher prevalence of mental health symptoms, such as anxiety, depression or apathy, which become more frequent compared to those with persistent COVID-19 who did not require hospitalisation during the acute phase. However, their analysis was limited to quantifying and comparing symptomatology. Rodriguez-Galan et al. [16] conducted a longitudinal study on HRQoL of post-COVID-19 patients and found that symptoms like low mood or anxiety were less prevalent during the acute stage of COVID-19 infection but increased in likelihood at three and twelve months post-diagnosis. However, their focus was solely on symptom description, with no correlation between symptom impact on HRQoL and time or progression since diagnosis. In line with previous research, our findings support the hypotheses regarding the emergence of such symptomatology. While our findings underscore significant sociodemographic and clinical variables associated with asthenia, low mood and anxiety in post-COVID-19 patients, it is crucial to acknowledge that these factors are not exclusive to the COVID-19 context.

Specifically, it is more likely that symptoms such as anxiety and depression develop over time rather than persist as primary symptoms of the disease in the long term, despite the presence of multiple overlapping causes in post-COVID-19 condition [13]. Our study highlights the significant prevalence and interrelation of asthenia, low mood and anxiety among post-COVID-19 patients. These symptoms often overlap in diagnostic criteria, suggesting common underlying causes and reflecting broader psychological distress, consistent with existing mental health research. These insights suggest that the observed mental health issues may also reflect general patterns of illness recovery, necessitating broader therapeutic approaches.

The selection of these variables was deliberate to capture the multifaceted nature of negative mental health in our sample. Asthenia is commonly observed in chronic illness phases, while low mood and anxiety are strongly correlated with physical symptoms, underscoring the interconnectedness of physical and mental health in long COVID-19 patients. These findings emphasise the need for a holistic approach in treating long COVID, addressing both physical and mental health symptoms.

### Limitations and Clinical Implications

Our study has several limitations. The sample size and the cross-sectional design may constrain the statistical power and the ability to establish associative relationships. It is important to note that the relationships observed in this study are associative and do not imply causation. Since the interviews captured information from patients at two different time points, caution is warranted to prevent potential measurement or recall biases. Nonetheless, we adhered to all relevant guidelines for observational studies [20] to mitigate bias to the greatest extent possible, and the interviews were conducted promptly relative to the participants’ previous situations. Moreover, this methodological approach is commonly employed during pandemic periods [34,35,36], and at the time of evaluation, national mobility restrictions were still in place. Although the need for technology could have been an exclusion factor, it was suitable for our middle-aged adult participants, for whom technology use is common. Asthenia, low mood and anxiety were recorded using a self-report questionnaire. Furthermore, in this study, data were collected only for asthenia, and future studies should be conducted to corroborate our findings using validated scales, as it would be interesting to analyse as an additional symptomatology. The inclusion of a control group would allow for a more robust comparison and help isolate the specific effects of COVID-19. Due to limitations in data availability and resources during the collection period, it was not possible to include an additional external control group. However, one of the strengths of our study lies in its ability to collect comprehensive data from individuals affected by persistent COVID. However, we believe that the pre–post measures provide valuable insight into the specific effects of post-COVID-19 syndrome, as each participant acts as their own control. Consequently, our study facilitated a thorough examination of the impact of sociodemographic variables, physical dimensions related to HRQoL and dependency status on the neurological clinical outcomes. These findings can inform healthcare professionals in making informed decisions regarding the prioritisation and management of affected individuals, enabling the implementation of more effective intervention programs aimed at enhancing patient functionality. Finally, some variables have not been considered in the present research and may show a relationship, such as level of education, comorbidities or medication intake. Further research and testing is still needed to corroborate and generalise our findings and their impact on this post-COVID-19 population.

## 5. Conclusions

The findings of this study suggest that, for individuals grappling with post-COVID-19 condition, being female and approaching the age of 50 are factors that increase the likelihood of experiencing mental health symptoms. Conversely, maintaining independence in daily activities and possessing good physical functioning serve as protective factors against the onset of mental health symptoms. Furthermore, a longer duration since diagnosis appears to exacerbate the prevalence and manifestation of symptoms such as fatigue, low mood and anxiety, perpetuating a diminished quality of life and compromised emotional well-being among patients. Our findings, while pertinent to post-COVID-19 patients, also align with broader patterns of negative mental health observed in the general population. This indicates that the mental health challenges identified may not be unique to the COVID-19 condition but, rather, part of a larger spectrum of post-illness recovery issues. Recognising this broader context is essential for developing effective treatment strategies that can be applied to both COVID-19 and other illness-related mental health issues, ensuring comprehensive patient care. Future longitudinal studies should support these findings, as well as the long-term follow-up of patients affected by post-COVID-19 condition.

## Figures and Tables

**Table 1:** Description and effect of the demographic and clinical variables in the prediction of asthenia. Current Long COVID-19 status.

	Asthenia		Univariate LR *		Multivariate LR *	
	No (n = 10)	Yes (n = 112)	OR * (95% CI *)	*p*-Value	OR (95% CI *)	*p*-Value
**Sex**, n (%)						
Male	4 (40.0)	23 (20.5)	1		1	
Female	6 (60.0)	89 (79.5)	2.58 (1.07–6.20)	**0.034**	6.45 (1.35–30.76)	**0.019**
**Age**, mean (SD) *	43.5 (5.8)	45.2 (6.2)	1.13 (1.00–1.27)	**0.042**	1.82 (1.50–2.20)	**<0.001**
**Time evolution**, mean (SD)	10.80 (2.39)	10.88 (3.41)	1.01 (0.83–1.22)	0.939	1.17 (0.88–1.55)	0.273
ADLQ * Total, mean (SD)	3.50 (2.17)	5.76 (2.22)	1.11 (1.04–1.19)	**0.002**	1.12 (0.98–1.29)	0.103
Physical_Functioning *, mean (SD)	53.50 (31.18)	25.18 (17.56)	0.96 (0.93–0.98)	**<0.001**	0.82 (0.72–0.94)	**0.004**
Role Physical *, mean (SD)	0.07 (0.01)	5.58 (17.66)	1.45 (0.94–2.22)	0.090	1.39 (0.94–2.04)	0.098
Bodily Pain *, mean (SD)	48.00 (19.29)	35.49 (22.05)	0.98 (0.95–1.00)	0.090	1.01 (0.96–1.05)	0.761
General Health *, mean (SD)	42.50 (23.60)	28.35 (15.01)	0.96 (0.93–0.99)	**0.014**	1.01 (0.95–1.06)	0.836

* LR: logistic regression. SD: standard deviation. OR: odds ratio. CI: 95% confidence interval. Level of dependency Activities Daily Questionnaire (ADLQ). SF-36 subscale scores of the physical component summary (PCS). The bold data has a significant meaning, *p* < 0.05.

**Table 2:** Description and effect of the demographic and clinical variables in the prediction of low mood. Current Long COVID-19 status.

	Low Mood		Univariate LR *		Multivariate LR *	
	No (n = 52)	Yes (n = 70)	OR * (95% CI *)	*p*-Value	OR (95% CI *)	*p*-Value
**Sex**, n (%)						
Male	15 (28.8)	12 (17.1)	1		1	
Female	37 (71.2)	58 (82.9)	1.96 (0.83–4.65)	0.127	1.61 (0.63–4.13)	0.318
**Age**, mean (SD *)	43.9 (5.3)	43.3 (6.2)	0.98 (0.92–1.05)	0.615	0.97 (0.90–1.04)	0.363
**Time evolution**, mean (SD)	9.61 (3.34)	12.23 (3.32)	1.06 (1.01–1.11)	**0.028**	1.32 (1.08–1.62)	**0.007**
ADLQ * Total, mean (SD)	5.67 (2.17)	4.44 (2.46)	0.96 (0.93–1.00)	0.057	0.93 (0.89–0.98)	**0.011**
Physical_Functioning *, mean (SD)	30.67 (20.79)	25.14 (19.93)	0.99 (0.97–1.01)	0.146	1.00 (0.98–1.03)	0.851
Role Physical *, mean (SD)	7.21 (18.75)	3.57 (15.51)	0.98 (0.96–1.00)	0.056	0.95 (0.91–0.99)	**0.013**
Bodily Pain *, mean (SD)	41.01 (23.03)	33.18 (20.81)	0.98 (0.97–1.00)	0.056	0.99 (0.97–1.01)	0.228
General Health *, mean (SD)	31.44 (17.16)	28.07 (15.47)	0.99 (0.97–1.01)	0.237	0.93 (0.89–0.97)	**0.001**

* LR: logistic regression. SD: standard deviation. OR: odds ratio. CI: 95% confidence interval. Level of dependency Activities Daily Questionnaire (ADLQ). SF-36 subscale scores of the physical component summary (PCS). The bold data has a significant meaning, *p* < 0.05.

**Table 3:** Description and effect of the demographic and clinical variables in the prediction of anxiety. Current Long COVID-19 status.

	Anxiety		Univariate LR *		Multivariate LR *	
	No (n = 52)	Yes (n = 70)	OR * (95% CI *)	*p*-Value	OR (95% CI *)	*p*-Value
**Sex**, n (%)						
Male	20 (29.4)	7 (13.0)	1		1	
Female	48 (70.6)	47 (87.0)	2.75 (0.87–8.67)	0.083	2.08 (0.75–5.79)	0.159
**Age**, mean (SD *)	43.2 (5.6)	44.5 (6.1)	1.06 (0.99–1.13)	0.078	1.55 (1.11–2.18)	**0.011**
**Time evolution**, mean (SD)	10.26 (3.38)	11.81 (3.28)	1.10 (0.99–1.23)	0.075	1.22 (1.12–1.32)	**<0.001**
ADLQ * Total, mean (SD)	6.50 (2.49)	5.14 (2.04)	1.03 (1.00–1.07)	0.068	0.91 (0.85–0.98)	**0.011**
Physical_Functioning *, mean (SD)	31.03 (21.22)	23.06 (18.57)	0.98 (0.96–1.00)	**0.046**	0.88 (0.78–0.99)	**0.034**
Role Physical *, mean (SD)	6.99 (20.19)	2.78 (11.56)	0.98 (0.96–1.01)	0.190	0.99 (0.96–1.01)	0.304
Bodily Pain *, mean (SD)	38.71 (22.60)	33.75 (21.20)	0.99 (0.97–1.01)	0.218	0.99 (0.97–1.01)	0.463
General Health *, mean (SD)	29.19 (16.99)	29.91 (15.37)	1.00 (0.98–1.03)	0.808	1.02 (0.99–1.06)	0.170

* LR: logistic regression. SD: standard deviation. OR: odds ratio. CI: 95% confidence interval. Level of dependency Activities Daily Questionnaire (ADLQ). SF-36 subscale scores of the physical component summary (PCS). The bold format has a significant meaning, *p* < 0.05.

## Data Availability

The data presented in this study are available on request from the corresponding author.
